# Effect of Argon on the Properties of Copper Nitride Fabricated by Magnetron Sputtering for the Next Generation of Solar Absorbers

**DOI:** 10.3390/ma15248973

**Published:** 2022-12-15

**Authors:** C. A. Figueira, G. Del Rosario, D. Pugliese, M. I. Rodríguez-Tapiador, S. Fernández

**Affiliations:** 1 Department of Applied Mathematics, Materials Science and Engineering and Electronic Technology, Higher School of Experimental Sciences and Technology (ESCET), Rey Juan Carlos University (URJC), C/Tulipán s/n, 28933 Móstoles, Spain; 2 Energy Department, Energy, Environmental and Technological Research Centre (CIEMAT), Av. Complutense 40, 28040 Madrid, Spain; 3 Technology Support Center CAT, Rey Juan Carlos University, C/Tulipán s/n, 28933 Móstoles, Spain; 4 Department of Electronics and Telecommunications (DET), Politecnico di Torino, 10129 Turin, Italy; 5 Department of Applied Science and Technology (DISAT) and RU INSTM, Politecnico di Torino, 10129 Turin, Italy

**Keywords:** copper nitride, reactive RF magnetron sputtering, gaseous environment, solar absorber

## Abstract

Copper nitride, a metastable semiconductor material with high stability at room temperature, is attracting considerable attention as a potential next-generation earth-abundant thin-film solar absorber. Moreover, its non-toxicity makes it an interesting eco-friendly material. In this work, copper nitride films were fabricated using reactive radio frequency (RF) magnetron sputtering at room temperature, 50 W of RF power, and partial nitrogen pressures of 0.8 and 1.0 on glass and silicon substrates. The role of argon in both the microstructure and the optoelectronic properties of the films was investigated with the aim of achieving a low-cost absorber material with suitable properties to replace the conventional silicon in solar cells. The results showed a change in the preferential orientation from (100) to (111) planes when argon was introduced in the sputtering process. Additionally, no structural changes were observed in the films deposited in a pure nitrogen environment. Fourier transform infrared (FTIR) spectroscopy measurements confirmed the presence of Cu–N bonds, regardless of the gas environment used, and XPS indicated that the material was mainly N-rich. Finally, optical properties such as band gap energy and refractive index were assessed to establish the capability of this material as a solar absorber. The direct and indirect band gap energies were evaluated and found to be in the range of 1.70–1.90 eV and 1.05–1.65 eV, respectively, highlighting a slight blue shift when the films were deposited in the mixed gaseous environment as the total pressure increased.

## 1. Introduction

Copper nitride (Cu_3_N) is attracting the attention of researchers due to its interesting properties, primarily its anti-ReO_3_ structure and its bandgap energy (*E_g_*) value. Its main attraction is that it is a non-toxic, metastable, and low-cost semiconducting material with earth-abundant elements and high stability at room temperature (RT). In addition, different studies have reported a wide range of band gap energy values, i.e., from 0.8 eV to 1.9 eV, which are easily achievable by controlling the deposition conditions [1,2]. The density functional theory (DFT) calculations, on the other hand, predict a relatively smaller *E_g_*, ranging from 0.23 eV to 1.0 eV [3]. In general, the materials required for photovoltaic cells are semiconductors with a band gap between 1.1 eV and 1.7 eV [4]. These values are necessary to promote an electron from the valence band to the conduction band, thus generating an electric current. In the case of Cu_3_N, the reported *E_g_* values suggest that this material can be a potential solar absorber for the next generation of photovoltaic devices [5]. In this sense, Cu_3_N has garnered interest in different application fields, such as optical storage media [6], tunnel junctions [7], solar energy conversion [8], and photovoltaics [9], due to its unique crystal structure and physicochemical properties. 

**Figure 1:**
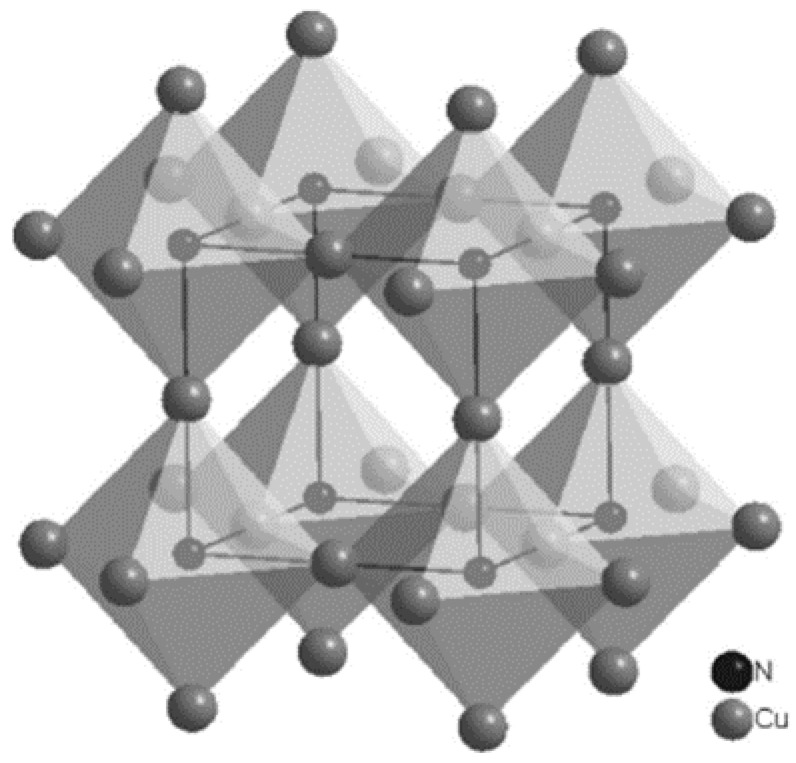
The cubic anti-ReO_3_ crystal structure of Cu_3_N [4].

Cu_3_N exhibits a cubic anti-ReO_3_ structure. The cell structure is shown in Figure 1. 

The ideal crystal structure shows a cationic octahedral complex with an anion in the center, where the N atom occupies the Wyckoff position 1a (0, 0, 0) and the three Cu atoms are located at the 3d Wyckoff positions (½, 0, 0; 0, ½, 0; 0, 0, ½). In addition, in this structure, a void can be found at the body center (½, ½, ½) position [10]. The experimental lattice constant of cubic Cu_3_N ranges from 0.3815 nm to 0.3885 nm [11]. Since Cu_3_N is a non-stoichiometric interstitial compound, its lattice constant can be easily adjusted by varying the process conditions and/or doping with other atoms. Several studies have shown that the incorporation of transition metal atoms (e.g., Pd) generates a decrease in the energy gap value and increases the material’s electrical conductivity [12,13].

Cu_3_N films have been successfully fabricated using various methods, such as chemical vapor deposition (CVD) [14], pulsed laser deposition (PLD) [15], atomic layer deposition (ALD) [14], and reactive radio frequency (RF) magnetron sputtering [16]. Among these, sputtering deposition is highly preferable because it can be performed at low cost, without the use of toxic gases, and with low energy consumption because it can be carried out at RT. 

Regarding the photovoltaic (PV) sector, Cu_3_N has been extensively studied and has been proposed as a possible substitute for silicon, which is the most widely used material in solar panels. In addition, thanks to its good electrical properties and the ease with which it can be fabricated, the use of this material as a solar absorber could help avoid the high-temperature steps involved in the fabrication of solar-grade silicon. Furthermore, thanks to bipolar doping [17], this material can be profitably used in rectifying heterojunctions [18,19,20] as well as homojunctions [8,9,10,11,12,13,14,15,16,17,18,19,20,21], and in solar cells [8,17,22]. 

In this work, the impact of using argon (Ar) in the RF magnetron sputtering process on all the properties of the Cu_3_N thin films fabricated on glass at RT have been thoroughly evaluated. In the material deposition, two different gaseous environments were used: an Ar-free environment based on pure nitrogen (N_2_) and an environment consisting of a changeable mix of N_2_ and Ar. Our main aim was to determine how the presence of Ar in the sputtering chamber might modify the films’ properties. Within this framework, it is of paramount importance to establish the sputtering conditions under which the material could be considered more suitable to be used as a solar absorber in a photovoltaic device.

## 2. Materials and Methods

The Cu_3_N thin films were deposited on silicon and glass substrates using reactive RF magnetron sputtering in a mono chamber (a commercial MVSystem). In this system, the RF-operated cathode was vertically adjustable, and the substrate was placed in front of the metallic target, a 99.99% Lesker Cu 3-inch diameter disk. The substrates had been prepared prior to the sputtering deposition. The silicon wafers were immersed in a hydrofluoric acid solution for 3 min and then ultrasonically cleaned for 3 min in a distilled water solution. The glass substrates were ultrasonically cleaned for 3 min in ethanol and distilled water solutions. The substrates were then dried by blowing nitrogen gas and loaded into the sputtering chamber. The base pressure of the chamber was around 2.6 × 10^−5^ Pa, and the distance between the target and the substrate was set at 98.8 mm. A pre-sputtering process to clean the target surface was carried out for 10 min using the sputtering conditions chosen for the deposition. The deposition time was set at 30 min and the films were deposited at RT and 50 W of RF power. The process gases used were N_2_ (99.999%), with a flow rate of 20 sccm, and Ar (99.99995%), with a flow rate of 10 sccm, both controlled by MKS mass flow controllers (MFCs) (MKS Instruments, Andover, MA, USA). The partial pressure of the N_2_, defined as R = [N_2_]/([N_2_] + [Ar]), was set at 0.8 and 1.0, and the total gas flow was maintained at 30 sccm. The total pressure in the chamber was varied from 3.5 Pa and 5.0 Pa by adjusting the position of the “butterfly” valve inside the magnetron system. 

The film thickness was measured with a model Dektak 8 profilometer (Bruker, San José, CA, USA). For all samples, a tip force of 68.67 µN and a scan size of 2000 µm were used. The crystallinity of the Cu_3_N films was determined by X-ray diffraction (XRD) using a commercial system (model PW3040/00 X’ Pert MPD/MRD) (Malvern Panalytical Ltd., Malvern, UK) and Cu-kα radiation (λ = 0.15406 nm). The scanned 2*θ* range was 10–60°, with a step size of 0.01° and a time per step of 20 s. Ultra-high resolution field emission scanning electron microscope (FESEM) NovaNanoSEM 230, (FEI-company, Hillsboro, OR, USA) was used to determine the variation in the surface morphology as a function of the gaseous environment used. To enable the FESEM analysis, the samples had previously been covered with a thin platinum layer (2 nm). The FESEM images were taken at 30 kX, 60 kX, and 120 kX. This system was also equipped with an EDAX Apollo X silicon drift detector energy dispersive X-ray spectroscopy (EDS) system (EDAX Meter company, Leicester, UK). Several regions on the surfaces were analyzed at an acceleration voltage of 10 kV to qualitatively quantify the amount of Cu L_α_, N K_α_, and O K_α_. Atomic force microscopy (AFM) (multimode nanoscope AFM model III, SPM, Veeco-Digital instrument Inc., Aschheim/Dornach Munich, Germany) was performed to investigate the surface of the specimens. The AFM measurements were carried out in tapping mode using silicon nitride AFM tips (OTR8, Veeco). The roughness was quantified by root mean square (RMS) in 1 × 1 μm^2^ two-dimensional (2D) micrographs. The chemical composition of the Cu_3_N thin films was characterized with Fourier transform infrared (FTIR) spectroscopy (Waltham, MA, USA) using a commercial Perkin Elmer Spectrum 100. The spectra were acquired using the transmittance mode (%) vs. the wave number, in the range of 400–4000 cm^−1^. A surface analysis was carried out with X-ray photoelectron spectroscopy (XPS) using a PHI 5500 Multitechnique System (Physical Electronics^®^ GmbH, Feldkirchen, Munich, Germany) and a monochromatic Al K-alpha X-ray source (1486.74 eV). This system was calibrated with the 3d_5/2_ line of Ag, perpendicular to the analyzer axis. The XPS spectra were analyzed with a Shirley background type and Gaussian–Lorentzian overlapping made with specific software. The data were fitted from the Cu-2p, N-1s, and O-1s states.

Finally, the optical transmittance was measured with a commercial Perkin Elmer Lambda 1050 spectrophotometer (Waltham, MA, USA) at normal incidence and RT and in the wavelength range of 400–2500 mm. The optical energy band gap was determined using the curve of (*αhν*)*^p^* vs. photon energy, *hν*, by extrapolating the line to the abscissa of *hν*, according to the standard Tauc plot [23]. The index *p*, which characterizes the type of electronic transition, has specific values of 2 and 1/2 for the indirect and direct transitions, respectively. The refractive index was also evaluated in the near-infrared (NIR) region at the wavelengths of 825 nm, 1061 nm, 1312 nm, and 1533 nm, using the prism coupling technique (2010, Metricon Corporation, Pennington, NJ, USA). Ten scans were performed for each measurement and the estimated error of the measurement was ±0.001.

## 3. Results

Table 1 shows the data for the sputtering parameters used in the Cu_3_N films deposition.

Two different behaviors were observed, depending on the process gas used. In the case of the sputtering deposition in the Ar + N_2_ gas mixture, the rate decreased with the total pressure, as previously reported in the literature [17,24,25]. This effect can be ascribed to the increase in the number of collisions that the atoms in the plasma undergo when the total pressure rises. In this scenario, the mean free path of the atoms present in the plasma was clearly reduced, decreasing the deposition rate. On the other hand, the higher deposition rate observed in sample S2 was attributed to a poorer nitridation because of the lower pressure used and, hence, the deposition was performed in a more metallic regime [25,26].

In contrast, the deposition rate obtained when the sputtering process was performed in a pure N_2_ environment revealed to be almost constant at the total pressure range used. Taking into account the fact that, in this case, the N atoms directly reacted with the Cu atoms from the target, the fact that no change in the deposition rate was obtained with the total pressure could indicate that a target “poisoning” effect might have occurred in the samples S3 and S4. This impeded the performance of the reactive magnetron sputtering, resulting in a significantly reduced sputtering yield and, hence, no increase in the deposition rate was observed [26].

**Figure 2:**
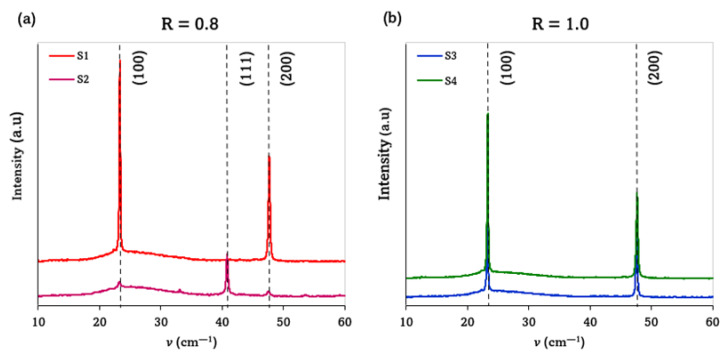
X-ray diffraction patterns of Cu_3_N films deposited at RT and 50 W in (**a**) mixed Ar + N_2_ and (**b**) pure N_2_ environments.

Figure 2a shows the XRD patterns of the Cu_3_N films deposited at the total pressures of 3.5 Pa and 5.0 Pa in a gas mixture of N_2_ + Ar. As can be seen, the sample deposited at the total pressure of 5.0 Pa presented the (100) plane as the preferential orientation (2*θ* = 23.33°), which is typical for N-rich thin films [19]. Another diffraction peak of lower intensity also appeared, corresponding to the (200) plane. In contrast, the sample deposited at the total pressure of 3.5 Pa presented the (111) plane (2*θ* = 40.79°) as the preferential orientation, indicative of a Cu-rich phase [19]. In addition, other diffraction peaks, such as (100), (110), (200), and (210), appeared less intensely, and the appearance of the (100) plane, indicating an N-rich phase, suggests that the material continued to be non-stoichiometric [27]. According to reports in the literature [21], when sputtering deposition is carried out in a mixed gas atmosphere, the amount of N_2_ at low pressure regimes is scarce, so Cu-rich (111) planes are more likely to form in the film. By increasing the total working pressure, the amount of N_2_ in the chamber begins to increase slightly, and may be high enough to form N-rich (100) planes.

Figure 2b shows the XRD patterns of the Cu_3_N films deposited at the total pressures of 3.5 Pa and 5.0 Pa in a pure N_2_ atmosphere. Regardless of the total pressure, the patterns exhibited both the (100) and (200) diffraction planes of the Cu_3_N structure, without the appearance of other planes [28]. This supports the idea of a possible target “poisoning” effect at total pressures higher than 3.5 Pa, which is in agreement with the absence of variation observed in the deposition rate for these samples [25,26]. 

Moreover, all the samples presented a cubic anti-ReO_3_ structure, and the absence of impurity peaks (such as Cu or CuO_x_) was indicative of a successful Cu nitridation to achieve Cu_3_N thin films with a high degree of purity [28]. 

The lattice constant (1) was calculated according to the interplanar spacing using Bragg’s law (2):

(1) $$d_{hkl}=a/\sqrt{h^{2}+k^{2}+l^{2}}$$

(2) $$d_{hkl}=\frac{n\lambda }{2\sin\theta },$$

where *d_hkl_* is the interplanar spacing of Miller indices *h*, *k*, and *l*, *n* is the order of diffraction, *λ* is the wavelength of X-rays, and *θ* is the diffraction angle. The theoretical value of the Cu_3_N lattice constant used in the calculation is 0.3815 nm [5,27]. The averaged grain size was also determined by using Debye–Scherrer’s Equation (3):

(3) $$\tau =\frac{k\lambda }{\beta \cos\theta },$$

where *τ* is the estimated grain size of the films, *β* is the full width at half maximum (FWHM) of the corresponding diffraction peak, *θ* is the diffraction angle, *λ* is the X-ray wavelength (0.15406 nm), and *k* is a constant.

Table 2 summarizes the parameters such as FWHM, lattice constant, and grain size calculated from the XRD patterns showed in Figure 2. The FWHM value obtained for the sample S2, deposited at the total pressure of 3.5 Pa in the Ar + N_2_ environment, was significantly higher than that of the counterpart sample S4, deposited in a pure N_2_ atmosphere. This could indicate a superior quality of the Cu_3_N films when deposited without introducing Ar into the process gas. Apart from the sample S4, the lattice parameters obtained were larger than the theoretical ones, which could indicate an over-stoichiometry. Specifically, this state consisted of additional N atoms inserted into the Cu_3_N lattice, probably as interstitials, revealing the formation of defects that could play an important role in increasing the lattice parameters [29]. This finding also agrees with the higher FWHM values obtained for the samples with larger lattice parameters. In all cases, these values are very close to that found in the literature for stoichiometric films fabricated by magnetron sputtering, i.e., 0.3815 nm [12,21,28,29,30,31].

All the Cu_3_N layers showed a single phase, with grain size values ranging from 29 nm to 45 nm, the grains of higher size being found in the samples with lower FWHM values. This would corroborate once again the better crystal quality of the samples deposited in the pure N_2_ atmosphere.

**Figure 3:**
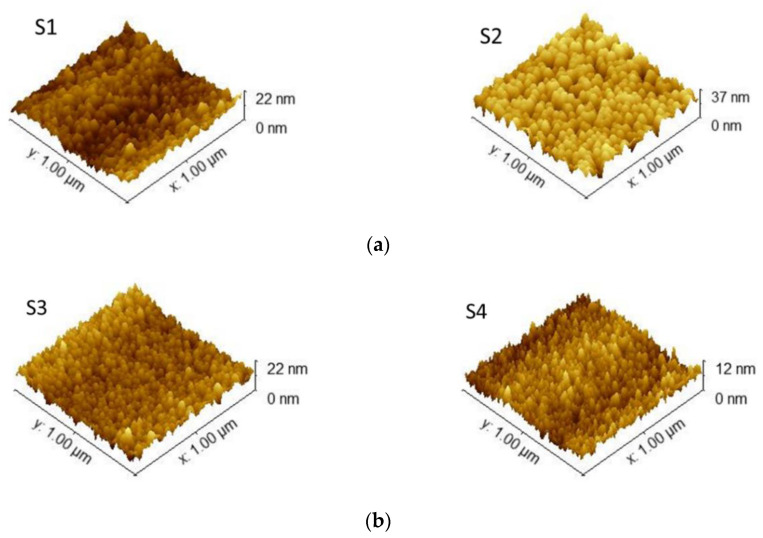
Three-dimensional micrographs (1 × 1 µm^2^) of the Cu_3_N films deposited in (**a**) N_2_ + Ar mixture and (**b**) pure N_2_ environments.

The surface morphology of the samples was analyzed using AFM. Figure 3 depicts the 1 × 1 µm^2^ 3D micrographs of the sputtered films fabricated in the N_2_ + Ar environment (Figure 3a) and in the pure N_2_ atmosphere (Figure 3b).

All the films presented a granular structure with tightly packed columnar grain features and sharp grain boundaries. The surfaces were smooth and uniform, regardless of the sputtering atmosphere used. However, two different morphologies were observed, depending on the composition of the process gas. This effect can be attributed to the increase in the deposition rate due to the introduction of Ar into the plasma, and also to the competition between the grain orientations present in the films, which led to changes in the morphology. Hence, the samples deposited in the N_2_ + Ar environment (Figure 3a) showed a large number of void boundaries embedded in a complex pyramidal conical morphology, indicative of a competition between the (111) and (100) orientations, as revealed by the XRD patterns (Figure 2) [32]. The RMS values calculated for these samples were 3.03 nm (S1) and 5.12 nm (S2). A rougher surface was obtained for the sample S2 deposited at the total pressure of 3.5 Pa. Under such a pressure value, this sample would be deposited in a more metallic regime, where a lower number of N_2_^+^ ions were reacting with the sputtered Cu atoms [25,26]. For this reason, its XRD pattern showed a competition between the grain orientations that led to a change in the preferred orientation, as was observed previously.

On the other hand, the films prepared in the pure N_2_ atmosphere showed a relatively more compact surface, almost without the presence of voids. Under these conditions, more N_2_^+^ ions had the ability to combine with the Cu atoms, thus resulting in a high-density film with a smoother surface. In this case, the RMS values were very similar: 1.3 nm (S3) and 2.2 nm (S4). However, a slight reduction was observed when the sample was deposited at the highest deposition pressure used in this work. It is worth noting that the smoother surfaces exhibited by the films deposited at higher pressures was a measurable effect independent of the environment used and could be ascribed to the higher energy of the ion bombardment on the substrate [31]. Hence, it is clear how the presence of Ar in the process gas determined the type of surface morphology. The surface morphology and the roughness are parameters that must be taken into account when manufacturing a device. 

**Figure 4:**
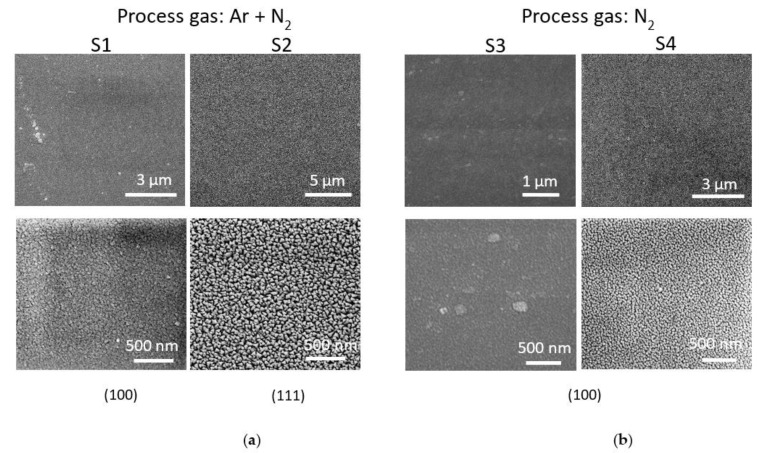
FESEM images of the surface of the Cu_3_N samples deposited in (**a**) N_2_ + Ar and (**b**) pure N_2_ environments. Information concerning the preferred orientation extracted from the XRD analysis is also included.

Figure 4 presents plan-view FESEM images of the Cu_3_N films deposited in the N_2_ + Ar environment (Figure 4a) and in the pure N_2_ atmosphere (Figure 4b).

These images confirmed that the surfaces were smooth and uniform, and mainly composed of columnar grains typical of the sputtering method [33]. Moreover, these results are consistent with those obtained from the previously reported AFM analysis. As can be observed, the grain shapes were influenced by the environment and by the total pressure used in the deposition. A pyramidal conical structure was observed in the films deposited at the lowest pressure of 3.5 Pa, and this was considerably oversized when the film was deposited in the N_2_ + Ar environment. The shape of these grains turned out to be more spherical when the sample was deposited in a pure N_2_ atmosphere. On the other hand, at the highest pressure of 5.0 Pa, the grains on the surface appeared to gain a nodular-like morphology, and this was once again oversized on the surface of the sample deposited in the mixed environment [32]. This morphology was characterized by the presence of a flat block of particles of uniform size, without visible cracks. These results are in line with the roughness calculated from the AFM images because these samples showed considerably smooth surfaces. The achievement of compact films would be beneficial for the production of heterojunctions needed for solar devices. 

The EDS data, shown in Table 3, revealed a qualitative ratio of Cu to N lower than three and, hence, the obtained films did not become a stoichiometric material, being N-rich in all cases. From these measurements, the samples S1 and S2, deposited in the gas mixture, showed a very similar ratio, being within the confidence interval of less than 2%, whereas the sample S4, deposited at the pressure of 3.5 Pa, showed a lower ratio than S3, which was attributed to the lower presence of N in the plasma used to form the nitride film. However, the Cu/N ratios obtained for the samples deposited in the N_2_ + Ar atmosphere were higher than those measured for the samples deposited in the pure N_2_ environment. This can be explained by the higher number of N_2_^+^ ions present in the pure atmosphere in comparison with the mixed one at the same pressure. Finally, the presence of O on the surface was observed in all cases. 

To confirm the chemical compositions of the Cu_3_N films, X-ray photoelectron (XPS) measurements were carried out. The XPS spectra showed the photoelectron emissions from the Cu-2p, N-1s, and O-1s states. The data obtained are summarized in Table 4. The presence of O was also observed in this case, as in EDS measurements. The large amount of O detected can be explained by surface contaminations with C and O and, hence, by the appearance of C-C, C-O, and C=O bonds due to a long exposure to ambient air, as other authors have pointed out before [29]. It should be noted that the presence of O was not observed in the XRD spectra, which could confirm that the formation of such oxidized compounds would take place on the surface and that they would have an amorphous character. The extracted Cu/N ratio for the samples deposited in the pure N_2_ environment was in the range of 1.46–1.56. In contrast, a slightly higher Cu/N ratio, ranging from 1.89 to 1.96, was obtained for the samples deposited in the N_2_ + Ar mixture. All these values were significantly smaller than the expected ratio of 3 corresponding to a stoichiometric Cu_3_N film. This would confirm the presence in all cases of the N-rich (100) plane in the XRD spectra, and also the increased incorporation of N in the samples deposited in the pure N_2_ environment, as previously revealed by the EDS data. 

**Figure 5:**
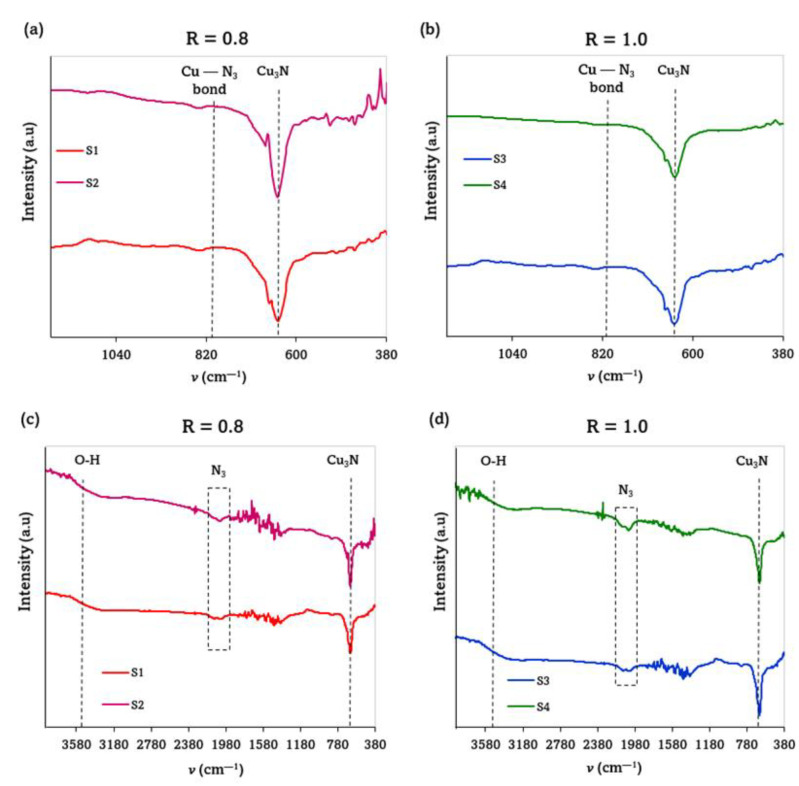
FTIR spectra of the Cu_3_N films deposited in (**a**,**c**) an N_2_ + Ar gas mixture and (**b**,**d**) a pure N_2_ atmosphere.

To further investigate the molecular structure, a FTIR analysis was carried out. As is shown in Figure 5a,b, all the samples exhibited a single band at around 644–648 cm^−1^, which confirmed the creation of the Cu–N bond. As other authors have reported, Cu_3_N exhibits a prominent peak at 652 cm^−1^, which can be ascribed to the intrinsic lattice mode vibration of Cu–N [30]. This would indicate that the amount of N was adequate to form the Cu_3_N phase. A weak peak at around 819 cm^−1^, assigned to the Cu–N_3_ bond, was also observed in all cases. In addition, a peak at 2049 cm^−1^ appeared in the whole spectra pictured in Figure 5c,d, corresponding to the stretching vibration of N_3_ azide and confirming the formation of an N-rich Cu_3_N material, in agreement with the data shown previously.

From Figure 5a, it is possible to highlight that the samples S1 and S2 deposited in the N_2_ + Ar environment presented a small shoulder at higher wavenumbers, i.e., at around 670 cm^−1^ and 690 cm^−1^, respectively, and that this was almost undetectable in the samples deposited in the pure N_2_ atmosphere. The presence of this additional band could be attributed to two effects: a possible oxidation of the surface sample (more probable in the samples S1 and S2, which showed voids in their morphology) and/or the formation of an incomplete Cu–N bond [34]. To confirm the presence of O in such high quantities as the EDS and XPS results suggest, the FTIR spectra were further analyzed at larger wavenumbers, as pictured in Figure 5c,d. As the whole spectra is revealed, the stretching vibrations of the hydroxyl group O-H can be observed in all the spectra in the broad region of 3775–3550 cm^−1^, regardless of the gaseous environment [35].

Table 5 summarizes the FWHM values and the wavenumber at which the center of the prominent peak was located, as obtained from the FTIR spectra. Once more, the samples deposited in the pure N_2_ atmosphere presented narrower peaks, and thus lower FWHM values, particularly the sample deposited at the lowest total pressure of 3.5 Pa, indicating its improved quality, in agreement with the extracted XRD results. In the case of the samples deposited in the N_2_ + Ar mixture, considering the main peak and the small shoulder as a single peak, the FWHM value was much higher for the sample S2, indicating a preferred orientation of (111) and poorer crystal quality (see Table 2).

**Figure 6:**
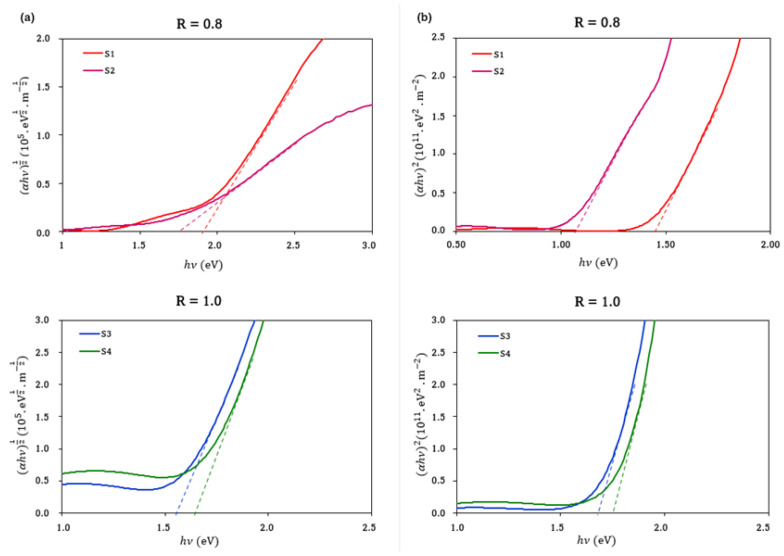
Calculation of the band gap energy of Cu_3_N films deposited at different pressures and in different gaseous environments: (**a**) (*αhν*)^1/2^ vs. *hν* plot for the determination of the direct band gap; (**b**) (*αhν*)^2^ vs. *hν* plot for the determination of the indirect band gap.

Finally, the effect of the sputtering environment on the optical properties of the samples was also evaluated. The band gap energy (*E_g_*) was determined from the transmission spectra using the standard Tauc plot [23]. The direct band gap was calculated by plotting (*αhν*)^1/2^ vs. *hν* and by extrapolating a full line to the abscissa of *hν* (Figure 6a). Similarly, the indirect band gap was calculated by plotting (*αhν*)^2^ vs. *hν* and extrapolating a full line to the abscissa (Figure 6b). The data obtained are summarized in Table 6.

The direct and indirect band gap values for the samples S1 and S2, deposited in the mixed N_2_ + Ar atmosphere, were found to vary within the range of 1.77–1.90 eV and 1.05–1.45 eV, respectively, depending on the total pressure. As the work pressure increased, a blue shift was observed. Xiao et al. [36] attributed this fact to a slightly higher number of Cu atoms being unable to combine with N atoms to form Cu–N bonds due to the relatively low number of N atoms present in the gas mixture. Such Cu atoms would act as a light-scattering center, forming defects which would lead to a decrease in the band gap energy. In this scenario, the energetic neutral N_2_^+^ ions in the plasma are dissociatively reflected by the target. This fact might account for a possible Cu re-sputtering, thus leading to a greater deficiency in the stoichiometric properties of Cu_3_N films, as revealed by the EDS and XPS data. 

On the other hand, the samples S3 and S4, deposited in the pure N_2_ atmosphere, showed direct and indirect band gap values in the range of 1.70–1.79 and 1.60–1.65, respectively. In this case, the opposite phenomenon was observed, namely the band gap energy increased when the gas pressure decreased [28]. This behavior can be explained by the different nature of the gaseous environment in which the deposition was carried out. At a pressure of 3.5 Pa, an excess of Cu atoms may have continued forming the material due to the higher number of N atoms in the pure N_2_ environment. However, when the N_2_ pressure increased, the excess N that did not form Cu–N bonds could have integrated into the lattice in interstitial places, resulting in a worsening of the structural quality, as shown by the XRD results. This would lead to the appearance of defects and, hence, to a decrease in the band gap energy [16,37]. In any case, the band gap energy values obtained were in the range considered suitable for solar absorber applications [38].

Finally, the refractive indices of the Cu_3_N films were measured at different wavelengths in the NIR region using the prism coupling technique. The obtained data are summarized in Table 7. As expected, the refractive indices showed a clear dependence on the measuring wavelength. Regardless of the gas environment used, the refractive index decreased with the wavelength, as reported in the literature [39].

These values were relatively low compared with those found in the literature [5,39,40]. This could be attributed to two factors: (i) the possible low density of the films due to the packed columnar grain features observed in the AFM images combined with the N-rich nature indicated by the EDS and XPS data; and (ii) the high O presence on the surface, determined from EDS and XPS data, leading to the formation of a very thin amorphous CuO_x_ film (not observable in the XRD patterns).

## 4. Conclusions

In this work, Cu_3_N films were prepared using reactive RF magnetron sputtering at RT at total pressures of 3.5 Pa and 5.0 Pa, and in different gaseous environments of N_2_ and N_2_ + Ar. The deposition rate calculated when the films were deposited in the mixture of N_2_ and Ar gases increased with the total pressure, while a constant deposition rate of 0.06 nm/s was obtained when pure N_2_ gas was used in the sputtering process, a fact attributed to a target “poisoning” effect. The XRD results revealed that the films had an anti-ReO_3_ structure. The samples deposited in the pure N_2_ environment showed the (100) plane as the preferential orientation. This plane corresponded to the N-rich phase of Cu_3_N, indicative of an increased presence of N atoms rather than Cu atoms within the plasma. However, a transition from the (100) to (111) planes as the preferential orientation was highlighted when the total Ar + N_2_ pressure decreased from 5.0 Pa to 3.5 Pa. At the same time, the FWHM values extracted from the XRD results revealed a worsening of the crystal quality when the films were deposited in the mixed gaseous environment. To conclude, a lattice constant larger than the theoretical one was obtained in any case, once again indicative of an N-rich phase. To support this hypothesis, FTIR, EDS, and XPS analyses were carried out, and the results confirmed the presence of an N-rich material and the formation of a Cu–N bond, regardless of the gaseous environment used. The XPS and EDS measurements also revealed a significant O contamination on the film surface, attributed to a long exposure in air. This contamination was also supported by the low refractive index values obtained for all of the films. Finally, the calculated optical band gap energies showed their strong dependence on the gaseous environment. In any case, a material with these values can be considered suitable for use as a solar absorber. However, all these results pointed to a preference for using (i) a pure N_2_ environment and (ii) a range of moderate pressures, such as 3.5 Pa, due to the better crystal quality of the films deposited.

## Figures and Tables

**Table 1:** Summary of the deposition conditions, the calculated deposition rate, and the measured thickness of the samples under study.

Sample	Total Pressure (Pa)	N_2_ Flux (sccm)	Ar Flux (sccm)	N_2_ Flow Ratio	Deposition Rate (nm/s)	Thickness (nm)
S1	5.0	20	10	0.8	0.05	96
S2	3.5	20	10	0.8	0.12	210
S3	5.0	20	--	1.0	0.06	115
S4	3.5	20	--	1.0	0.06	100

**Table 2:** Main structural parameters derived from the XRD measurements.

Sample	Total Pressure (Pa)	N_2_ Flow Ratio	Preferential Orientation	FWHM (°)	Lattice Constant (nm)	Grain Size (nm)
S1	5.0	0.8	(100)	0.1870	0.3817	42
S2	3.5	0.8	(111)	0.2755	0.3832	29
S3	5.0	1.0	(100)	0.1870	0.3820	42
S4	3.5	1.0	(100)	0.1771	0.3814	45

**Table 3:** Relative surface composition of the films under study, derived from the EDS analysis.

Sample	Total Pressure (Pa)	N_2_ Flow Ratio	Cu (at%)	N (at%)	Cu/N	O (at%)
S1	5.0	0.8	44.29	20.38	2.17	35.34
S2	3.5	0.8	53.52	25.46	2.15	21.06
S3	5.0	1.0	44.49	21.55	2.06	33.97
S4	3.5	1.0	38.26	19.67	1.94	42.07

**Table 4:** Relative surface composition of the films under study, derived from the XPS analysis.

Sample	Total Pressure (Pa)	N_2_ Flow Ratio	Cu (at%)	N (at%)	Cu/N	O (at%)
S1	5.0	0.8	31.63	16.68	1.89	51.69
S2	3.5	0.8	34.05	17.34	1.96	48.61
S3	5.0	1.0	28.01	19.15	1.46	52.84
S4	3.5	1.0	27.63	17.65	1.56	54.71

**Table 5:** Main parameters derived from the FTIR spectra. The FWHM values of the samples S1 and S2 have been estimated by considering the prominent peak and the small shoulder as a single peak.

Sample	Total Pressure (Pa)	N_2_ Flow Ratio	FTIR Peak (cm^−1^)	FWHM (cm^−1^)
S1	5.0	0.8	644	62.2
S2	3.5	0.8	645	80.3
S3	5.0	1.0	644	60.2
S4	3.5	1.0	648	58.9

**Table 6:** Direct and indirect band gap energy calculated for the samples under study.

Sample	Total Pressure (Pa)/ N_2_ Flow Ratio	Cu/N	Direct Band Gap (eV)	Indirect Band Gap (eV)
S1	5.0/0.8	1.89	1.90	1.45
S2	3.5/0.8	1.96	1.77	1.05
S3	5.0/1.0	1.46	1.70	1.60
S4	3.5/1.0	1.56	1.79	1.65

**Table 7:** Refractive indices of the samples under study measured at different wavelengths in the NIR region.

Wavelength (nm)/*hν* (eV)	S1	S2	S3	S4
825/1.503	1.521	---	1.522	1.512
1061/1.169	1.518	1.508	1.519	1.508
1312/0.945	1.515	1.506	1.515	1.506
1533/0.809	1.512	1.503	1.512	1.503

## Data Availability

Not applicable.
